# Neurological-Type Wilson Disease: Epidemiology, Clinical Manifestations, Diagnosis, and Management

**DOI:** 10.7759/cureus.38170

**Published:** 2023-04-26

**Authors:** Nathaniel Kipker, Kaitlyn Alessi, Marko Bojkovic, Inderbir Padda, Mayur S Parmar

**Affiliations:** 1 Foundational Sciences, Nova Southeastern University Dr. Kiran C. Patel College of Osteopathic Medicine, Clearwater, USA; 2 Foundational Sciences, Barry University, Miami, USA; 3 Internal Medicine, Richmond University Medical Center, New York, USA

**Keywords:** gene therapy, wilson disease, copper, atp7b, treatment, diagnostics, clinical manifestations, neurological type

## Abstract

Wilson disease (WD) is a complex metabolic disorder caused by disruptions to copper regulation within the body, leading to an unregulated accumulation of copper within various tissues. A less understood organ affected by the collection of copper is the brain, which further leads to the generation of oxygen-free radicals and resultant demyelination. Healthcare providers must keep the neurological form of WD in their list of differentials when patients present with diverse neurological manifestations. The initial step to diagnosis will be to distinguish the characteristic disease presentation with a thorough history and physical and neurological examination. A high clinical disease suspicion of WD should warrant further investigation by laboratory workup and imaging modalities to support the clinical findings and confirm the diagnosis of WD. Once a WD diagnosis is established, the healthcare provider should treat the underlying biological process of WD symptomatically. This review article discusses the epidemiology and pathogenesis of the neurological form of WD, its clinical and behavioral implications, diagnostic features, and treatment modalities (current and emerging therapies), further aiding healthcare professionals in early diagnosis and management strategies.

## Introduction and background

Wilson disease (WD) is a disorder of copper metabolism within the liver that has several systemic effects within the body. It is an autosomal recessive disorder that results in mutations of the ATPase copper-transporting beta *(ATP7B)* on chromosome 13 [1]. This gene is actively expressed within the liver, resulting in a lack of copper transport into bile and ceruloplasmin. This copper accumulation can affect the kidneys, liver, brain, and cornea [2]. If untreated, WD can result in liver cirrhosis and toxic effects on the brain due to unregulated copper accumulation.

The biological region where the copper accumulates in the human body determines its clinical manifestation and the type of WD. In the hepatic-type WD, the excess copper accumulates in hepatocytes, further affecting liver function. In contrast to the neurological-type WD, copper accumulates in the brain, resulting in neurological manifestations [1].

The excess copper accumulation and deposition are toxic to the central nervous tissue and can further lead to the generation of oxygen-free radicals, cell membrane damage, and reactive astrocytes. It is essential to maintain suspicion of the neurological-type WD by recognizing the key symptoms and characteristics that are manifested with disease onset and progression. Making a prompt diagnosis and initiating early treatment modalities can drastically improve the patient's disease course and prognosis and decrease the risk of complications.

Note: This article was previously posted to the preprint server available at Research Square (DOI: https://doi.org/10.21203/rs.3.rs-1754204/v1) on July 8, 2022.

## Review

Epidemiology

Mutations occurring in chromosome 13q14 disrupt the coding for the ATP7B protein resulting in dysfunctional copper excretion [3]. The disease can either develop sporadically or from an autosomal recessive inheritance pattern. It is a rare disease process that should be suspected in patients with a strong family history of consanguinity. Usually, symptoms begin during childhood or teenage years, manifesting as liver dysfunction [4]. As per Wilson Disease Association, WD affects approximately one in 30,000 people worldwide. In a study with 604 participants with WD, 47% had hepatic-type WD, and 32.2% had neurological-type WD [4]. Other types of WD manifestations can range from psychiatric to ophthalmic characteristics.

The prevalence of WD differs per geographical region and seems to predominate more in the Asian population than the Caucasian population [5]. Several regions statistically display higher rates of WD among their population, including Germany, Japan, and Austria [3]. However, the region with the highest incidence of WD in the world is Costa Rica, reporting a rate of 4.9/100000 inhabitants [3,6]. Their incidence rate is attributed to increased consanguinity within the country [3,6]. The most common mutation linked to WD in Europe and North America is p.H1069Q [5]. With this knowledge in mind, physicians can utilize genetic testing and take thorough family histories to reveal the diagnosis of WD.

Pathogenesis

An essential barrier in the human body is the blood-brain barrier. Normally, astrocytes can detoxify copper via a reduction-oxidation reaction, but the astrocytes reach their limited capacity with the dramatic increase in extrahepatic copper. This allows the copper to infiltrate the brain and damage the oligodendrocytes. Copper causes swelling of the myelin sheaths supplied by oligodendrocytes in the CNS, resulting in demyelination [7]. Along with the demyelination, copper deposits within certain brain regions like the basal ganglia, thalamus, cerebellum, upper brainstem, putamen, and frontal lobe [8]. Deposition causes mitochondrial damage, oxygen-free radical generation, white matter, and extracortical spinal tract damage. The widespread neurological dysfunction that copper causes makes it difficult to pinpoint and associate dysfunctional brain tissue with the presenting symptoms. Furthermore, since WD is extrahepatic, the diagnosis is often delayed. Another potential contributor to the neurological toxicity seen in WD is liver cirrhosis. This leads to portal hypertension, hepatic encephalopathy, and the decreased ability of the liver to detoxify neurological metabolites and excrete them properly [7].

Clinical manifestations

Tremor

The diagnosis of neurological-type WD is difficult due to the diverse neurological manifestations it can present with. The most notable symptom is a tremor which occurs in up to 55% of patients on the initial encounter and is present in about 90% of patients during the duration of the disease [6]. Contemporary literature has reported a delay of diagnosis from the onset of its first symptom by 2.5-6 years [8].

The pathology of WD is complex, making it challenging to diagnose based on the symptoms alone. Various forms of tremor can be noted, including resting, postural with "wing-beating" features, or kinetic [1,2]. Tremor severity progresses from proximal to distal. The regions affected are the pons, midbrain, thalamus, and putamen, with the latter being the most notable area affected in 81% of patients [1,9]. Like many other neurodegenerative diseases, the tremor can be unilateral or bilateral [1]. A study in 2018 analyzing the tremor of WD found that tremor asymmetry was influenced by the asymmetry of fiber volumes between the thalamus and cerebellum, as well as the caudate nucleus and the thalamus [10].

Therapeutic options vary to combat the specific type of tremor noted [7]. Non-selective beta-blockers, such as propranolol, are the first-line treatment for essential, postural, and kinetic tremors affecting the hands. Barbiturates, benzodiazepines, anticholinergics, presynaptic GABA agonists, botulism toxin injections, and even deep brain stimulation have been used in patients with symptomatic tremors [7].

Dystonia

Dystonia is the most severe and recurrent symptom of neurological-type WD [1]. It is seen in 11- 65% of patients with neurological-type WD and can be generalized, focal, or multifocal [2,7]. However, the most common dystonia appreciated in these patients is a focal one affecting the face. This is represented by an open mouth smile, a dystonic dropped jaw, or a fixed smile [2]. As the disease progresses, the dystonia transitions from focal to generalized, involving several different segments and resulting in a dystonic state associated with high mortality [2]. A study in 2019 looked at MRI with diffusion tensor imaging scans over 364 patients between the ages of 5 and 42 years that presented with neurological manifestations of WD. They found that out of the 364 patients, 197 had some form of dystonia. Of the 197 patients, the imaging displayed damage to the putamen at 80.7%, pons at 48.2%, and thalamus at 37.1% [9].

The treatment for dystonia in WD is also symptom-based. The multifocal or generalized form of dystonia can be treated with anticholinergics, GABA agonists, dopamine agonists, antiepileptic drugs, and botulism toxin injection. Usually, the botulism toxin injection is the first-line treatment for the focal form of dystonia. If pharmacotherapy is not showing improvement in symptoms, deep brain stimulation of the globus pallidus internus, pallidotomy, or thalamotomy may be the last resort alternatives [7].

Parkinsonism

Parkinsonism is reported in 19-62% of patients with neurological-type WD [7]. This syndrome includes rigidity, a resting tremor, bradykinesia, and postural imbalance [1,7]. Like many neurodegenerative diseases, the symptoms are often asymmetrical and can affect the signaling and induction of neurotransmitters. From a pathological standpoint, the development of parkinsonism in WD patients is related to copper accumulation and subsequent damage within the basal ganglia and substantia nigra.

The atypical signals found on MRI are likely caused by glial cell hyperplasia, necrosis, and edema [9]. Of 364 participants diagnosed with neurological-type WD, 127 reported parkinsonism symptoms [9]. Of the 364 participants, 81.9% displayed defects in the putamen, 32.3% in the caudate nucleus, and 26.4% in the pons seen on MRI [9].

Treatment includes levodopa and dopamine receptor agonists for patients with disabling symptoms. As disease severity progresses, deep brain stimulation or neuroablative lesions of the globus pallidus internus and subthalamic nucleus could be alternatives [7].

Cerebellar Ataxia

Cerebellar ataxia is a common symptom in neurological-type WD [1], with 30% of patients expressing some form of dysfunction [7]. The pathogenesis of cerebellar ataxia is related to the marked atrophy and loss of Purkinje cells in the cerebellum [11]. This leads to loss of balance, gait, and impairment in fine motor movements. If symptoms of WD do not accompany it, various other neurodegenerative disorders may be suspected, and the true diagnosis could be missed or delayed. Diagnosis of neurological-type WD is commonly made with cerebellar ataxia in conjunction with the other neurological manifestations described in this section [2]. Pharmacotherapy is not an option for patients with cerebellar ataxia; however, the increasing availability of genomics might lead to useful biologics that can prevent disease progression.

Choreoathetosis

Choreoathetosis is a symptom in 6-16% of WD patients, but it is not diagnostic of WD alone. The symptom is an involuntary twitching or writhing movement disorder that affects the head, trunk, and extremities unilaterally or bilaterally [2]. It was observed that out of 364 patients with neurological-type WD, only eight patients had associated choreoathetosis [9]. In all eight patients, the damage was seen in the caudate nucleus [9]. Another study in 2018 found that patients with choreic movement had marked asymmetry of the fiber volumes between the caudate nucleus and the thalamus [10]. The treatment of choreoathetosis is limited to dopamine depletion agents such as tetrabenazine and behavioral therapy.

Dysarthria

Dysarthria, or speech disturbance, is one of the most common neurological symptoms in WD patients [7]. The copper collection in the basal ganglia, cerebellar nuclei, corticobulbar nuclei, and their associated tracts causes several detrimental symptoms, including dysarthria [7]. It is suspected that the copper accumulation damages these cranial structures over time and leads to improper communication between the basal ganglia and the subthalamic nuclei [12]. As a result, WD patients may have difficulty controlling muscles involved with speech production and subsequent slurring. Dysarthria can be further subcategorized into specific types such as mixed unclassified, ataxic, dystonic, and hypokinetic [7]. Each type presents unique symptoms associated with the neurological structures involved. In the mixed unclassified-type dysarthria often presented in WD patients, the copper aggregation affects several structures, causing dystonic and hypokinetic characteristics and cerebellar ataxia [13]. Ataxic type dysarthria displays cerebellar symptoms, while dystonic and hypokinetic types express movement disabilities [7].

Treatment for dysarthria depends on the type and severity. Relaxation techniques can be encouraged in patients with dystonic type, and speech rate therapy can be utilized in cerebellar ataxic type dysarthria [7]. For the hypokinetic type, it may be beneficial to try loudness and articulation techniques [7]. Severe forms of dysarthria might require augmentative communication devices that can be accessed through smart devices [7]. It's imperative to treat dysarthria in WD patients; thus, they can experience a healthy lifestyle and feel like an active part of society, further increasing their quality of life.

Dysphagia

One of the most noteworthy symptoms found in WD patients is dysphagia [1]. It is defined as difficulty in swallowing, chewing, or oral transit and is present in 50% of patients with neurological-type WD [7]. Dysphagia can be due to impairment of muscle tone, an inability to coordinate movements, or even weak muscles involved in the process [7]. Clinically, patients may seem malnourished or experience drooling. Aspiration pneumonia is a deadly but common complication of dysphagia that should be prevented at all costs [14]. A clinician can determine if enteral feeding is necessary by completing a thorough physical examination and assessing a patient's nutritional status. Failure to provide enteral feeding in severe dysphagia cases dramatically increases the risk of aspiration pneumonia, malnutrition, and even mortality [14].

To prevent these fatal complications and preserve the quality of life in a patient with WD, physicians must consider appropriate therapies and follow up on time. For mild cases of dysphagia, regular checkups that analyze body weight measurements and nutritional status may be sufficient [7]. However, enteral feeding should be considered in high-risk patients to avoid serious complications. One case study exploring alternative treatment options indicated that neuromuscular electrical stimulation might be beneficial [14]. Additional research is necessary to confirm efficacious treatment for WD patients with dysphagia.

Behavioral and Psychiatric Manifestations

Approximately 30-40% of WD patients present with psychiatric symptoms at the time of diagnosis, which is essential to address [15]. Behavioral and psychiatric conditions secondary to WD can range from depression disorders to acute psychotic episodes [11]. These complications can be due to a patient's reaction to chronic disease or metabolic dysfunction. The copper levels damage the basal ganglia and hypothalamus, decreasing presynaptic serotonin transporters seen on single-photon emission computed tomography (SPECT) [15]. It is proposed that the chemical imbalance can then disturb mentality. Other studies have correlated mutations of *ATP7B* with specific personality characteristics, which could also explain these secondary disorders [15]. Some authorities also believe psychiatric and behavioral symptoms actually suggest severe WD and may potentially be a sign of irreversible brain damage [16]. The copper accumulation supposedly overcomes the liver's ability to function, which leads to hyperammonemia and hepatic encephalopathy [16].

Patients with secondary behavioral and psychiatric disorders due to neurological-type WD could manifest various symptoms. Behavioral symptoms include family problems, criminal actions, and abnormal verbal aggression [14]. A few reported conditions are mood disturbances, psychosis, sexual dysfunction, insomnia, and impaired social judgment. Since about 4-16% of WD patients attempt suicide, physicians should know the treatment options and schedule regular follow-ups to assess their mental health [15].

Treatment options are based on behavioral or psychiatric issues and should be used cautiously in neurological-type WD patients. Mood disorders are usually treated with tricyclic antidepressants, selective serotonin reuptake inhibitors, and serotonin-norepinephrine reuptake inhibitors. In severe cases, electroconvulsive therapy can be attempted [15]. Medications recommended for psychotic symptoms include olanzapine, clozapine, and quetiapine due to their efficacy in WD patients [15]. Another option that should be considered for most patients struggling mentally is cognitive-behavioral therapy and psychotherapy.

Since half of the patients diagnosed with neurological-type WD will have secondary psychiatric conditions, it is important to avoid antidepressants with a high risk of liver injury [11]. Physicians should avoid prescribing phenelzine, imipramine, iproniazid, amitriptyline, duloxetine, bupropion, and agomelatine due to their potential to further damage the liver in WD patients [15].

After beginning treatment, scheduling regular checkups with neurological-type WD patients is extremely critical. There is a potential to develop worsening psychiatric symptoms following therapy initiation, which demands immediate evaluation and treatment adjustment [15].

Other Characteristics

There are many other symptoms that neurological-type WD patients can manifest. Cognitive deficits that disrupt learning, working memory, and executive domains could be due to lesions in cortico-striatal pathways [15]. Restless leg syndrome, secondary neuropathies, headaches, new-onset tics, and myoclonus are other noteworthy symptoms of neurological-type WD [7]. Interestingly, generalized tonic-clonic seizures have been reported in 6% of WD patients and could be a critical warning of paradoxical worsening upon chelation treatment initiation [13].

Diagnostics

A timely diagnosis of neurological-type WD relies on high clinical suspicion, biochemical laboratory markers, histological assessment, and genetic findings [1]. Clinical suspicion for WD should be especially high if wing-beating tremor, dysarthria, drooling, dystonia, or atypical facial grimacing is observed [17]. One-third of patients may also present with psychiatric abnormalities [16]. In a study containing 268 subjects with psychiatric symptoms, 15 cases were reported due to a treatable underlying metabolic disorder such as WD [18].

After taking a thorough history and performing a complete neurological examination, a physician can proceed with the diagnostic algorithm for confirming neurological-type WD. A common scoring system that is used as a guideline and diagnostic tool for WD is the Leipzig score. The scoring system considers the presence of Kayser-Fleischer rings, neurological symptoms, urine copper levels, serum copper levels, and measured hepatic copper [19]. Depending on the copper laboratory values and the severity of the symptoms, the patient receives points that indicate the likelihood of disease. However, the Leipzig score was the most helpful for distinguishing hepatic-type WD from other hepatic pathologies [20].

The first laboratory test that should be ordered is serum ceruloplasmin, which is usually decreased by 50% in patients with WD [3]. A ceruloplasmin level of less than 20 mg/dL is usually consistent with a diagnosis of WD, but additional tests should be completed to rule out the possibility of false negatives [20]. Serum ceruloplasmin is considered an acute phase reactant and could potentially be elevated due to inflammatory states leading to false-negative results [3]. A low ceruloplasmin level of less than 0.1 g/L accompanied with Kayser-Fleischer rings on a slit lamp examination is considered to establish the diagnosis of WD [17]. The sensitivity and specificity of this laboratory test are recorded to be 95% and 84.5% for diagnosing WD [21]. In addition, the slit lamp examination can confirm the presence of Kayser-Fleischer rings if WD is suspected [3].

Measuring the 24-hour urinary copper excretion is the next step in the diagnostic algorithm. Adult patients with WD have levels that usually exceed 100 mcg/24 h, while pediatric patients typically have greater than 40 mcg/24h [3]. If the urinary copper excretion produces borderline values, it may be beneficial to do a D-penicillamine challenge [20]. The abnormal copper homeostasis displayed on laboratory tests tends to be most helpful in diagnosing neurological-type WD [20].

Other labs indicate WD is a serum-free copper greater than 200 mcg/L, but there are limitations in measuring direct copper levels [3]. Some new tools that aid in diagnosing WD include a radioactive copper ratio and a relative exchangeable copper test [19].

The gold standard for diagnosing WD is performing a liver biopsy which shows hepatic copper content greater than 250 ug/g dry weight [20]. A liver biopsy is only required to establish a diagnosis when the clinical presentation and laboratory tests fail to do so [K10]. At this point, clinicians might question using molecular analysis to aid diagnosis. Even though genetic tests may reveal a mutation in the *ATP7B* gene, they are impractical and could delay diagnosis [17].

Imaging techniques can also provide clues for diagnosing neurological-type WD but are not considered confirmatory tests. An MRI test can detect abnormalities in neurological-type WD, most commonly manifest as T2 hyperintensities within the putamen [1,20]. A case report of a woman with neurological symptoms was diagnosed with neurological-type WD and demonstrated the classic "panda sign" on a brain MRI [1]. The "panda sign" is seen when hyperintense signals are displayed around the midbrain, red nucleus, and substantia nigra [1].

The bright claustrum sign may also be appreciated as a thin rim displaying T2 hyperintensity within the claustrum [20]. A SPECT image can detect early brain damage that could be used as a guide for treatment purposes [17]. Magnetic resonance spectroscopy has exhibited decreased levels of N-acetylaspartate and N-acetylaspartylglutamate in neurological-type WD patients [20]. Medical imaging advances are very promising for upcoming diagnostic tools for WD.

The clinician should explore differential diagnoses, including chronic hepatitis, cirrhosis, and psychiatric disorders [17]. However, it is important to stress establishing a prompt diagnosis in WD patients to prevent permanent impairment. Unfortunately, the average time from symptom onset to diagnosis establishment remains approximately one year [20]. To avoid delay in diagnosis, physicians should maintain a high suspicion for neurological-type WD and follow a methodical diagnostic algorithm.

Current and emerging treatments

Chelation Therapy

Copper is an essential element within our body that contributes to various biological processes such as mitochondrial respiration, extracellular matrix cross-linking, antioxidant defense, and neurotransmitter biosynthesis [22]. Due to the defect in ATP7B, copper homeostasis is deregulated, and copper secretion is impaired. Chelation therapy introduces an element that binds to excess free copper, forms a ring-like structure, and promotes excretion. Copper is removed from tissues, and re-accumulation is prevented with chelation therapy. D-penicillamine (D-PCA), trientine, and dimercaptosuccinic acid (DMSA) are chelation therapies that promote copper excretion through the kidneys. These chelators can significantly improve liver injury and WD patient symptoms; however, they may have a limited effect on neurological symptoms. Tetrathiomolybdate (TTM), another chelator, promotes excretion through the gastrointestinal system [22]. Zinc salts are also used as an indirect chelator by decreasing copper absorption in the gastrointestinal system and inducing metallothionein, which promotes copper excretion in urine and feces [22].

D-PCA

D-PCA was first introduced in the 1950s as an oral chelator with great efficacy in mobilizing copper from tissues into the kidneys for excretion. The chemical makeup of D-PCA includes a thiol with a sulfhydryl group that can bind to copper and promotes its excretion via urine in WD patients [23]. D-PCA is indicated for initial therapy for symptomatic patients and long-term maintenance therapy [1]. There is a direct relationship between the reduction of copper stores within the body and the excretion of D-PCA; therefore, there is little fear of becoming copper-deficient [23]. The effect of this drug can be seen clinically with a reduction in neurological symptoms and a decrease in copper accumulation on MRI.

Lifelong treatment has been successful, but close monitoring is necessary due to the risk of paradoxical neurological worsening after beginning treatment. The mechanism of neurotoxicity is unclear; however, it is hypothesized that initial treatment mobilizes copper in the blood, accumulates in the brain, and causes free radical-induced tissue injury. The percentage of patients with worsening neurological symptoms is unknown and can range from 10% to 50%. It is proposed to start treatment at low dosages of D-PCA with the administration of vitamin E, which reduces free radicals to avoid the adverse effect of neurotoxicity [23]. Thus, clinically, monitoring the patient during the initial treatment is important. Relative contraindications of D-PCA include patients with severe neurological symptoms or liver failure [5].

Trientine (Triethylenetetramine)

Trientine was approved for patients that are intolerant of D-PCA. It is also a copper chelator that requires daily ingestion and has been shown to have increased urinary excretion of copper. It is also shown to be safer than D-PCA, more effective than zinc alone, and becoming a preferred treatment for WD. A once-daily dose shows the highest compliance for the drug with average tolerability [24]. However, the challenge with trientine is the very poor CNS penetration and, thus, the limited improvement of cerebral symptoms or even neurological deterioration [25]. There is emerging research to improve the delivery of trientine for improving their capability to enhance transfer across the BBB [25] and provide a beneficial clinical response to symptomatic neurologic WD patients.

TTM

TTM is a chelation therapy developed for patients with adverse reactions to D-PCA and trientine. Radiocopper studies have shown an immediate reduction in dietary copper reabsorption via the gut mucosa when TTM is given orally [23]. The mechanism of action involves TTM forming complexes with copper and proteins in the intestinal lumen, which promotes excretion through the biliary tract. Therefore, TTM is preferentially used in patients with renal complications [22]. TTM is more effective than zinc in copper chelation due to its immediate impact [23]. TTM has rarely been shown to cause neurotoxicity and has a short half-life with mild side effects [5]. Adverse effects like acute hepatitis, hypercholesterolemia, excessive triglycerides, and reversible bone marrow depression have been rarely reported [23]. It is important to know that prolonged therapy with TTM could be toxic due to the excess amounts of molybdenum [5].

Unfortunately, its clinical usage is reduced due to the high frequency of dosing, which can be up to six times a day [22]. Bis-choline-tetrathiomolybdate, a derivative of TTM, has recently been undergoing clinical trials to improve drug compliance. Bis-choline-tetrathiomolybdate has not displayed cases of paradoxical worsening for treating neurological-type WD [22].

DMSA

DMSA is a water-soluble analog of dimercaprol used in many heavy metal toxicities as an antidote. It can form complexes with copper ions, and oral sodium dimercaptosuccinate (Na-DMS) formulation significantly increases urinary copper excretion [26]. In China, DMSA was first used as a copper chelator for WD, and there are substantial experiences with the use of DMSA for WD treatment in China [26]. A study in 2020 found that when compared to D-penicillamine, DMSA could chelate copper from the brain faster and more effectively [27]. The improvement of neurological WD symptoms more quickly resulted in fewer aggravations than D-penicillamine [27]. However, there is no significant difference in long-term therapy [27]. Another study in 60 neurological WD patients observed that combination therapy of DMSA and zinc effectively improved the neurological symptoms of those with a history of neurological deterioration with D-penicillamine [28]. Over one to two years of treatment, 85% of patients showed improvement, 11.67% experienced a stable neurological condition, and 3.33% suffered deterioration of neurological symptoms [28].

Zinc

Zinc sulfate or zinc gluconate successfully inhibits copper absorption from the gut, effectively chelates copper, and facilitates excretion into the feces or urine [22]. Zinc is used for symptomatic patients that have previously taken copper chelators. In addition, some studies have proven zinc to be more effective in treating neurological-type WD than the hepatic type [23]. When zinc is given orally, it increases the production of metallothionein [23]. Metallothionein comprises cysteine-rich proteins with a strong affinity for copper; binding dietary copper, it blocks the absorption of copper in the intestinal tract [23]. In terms of preventing re-accumulation of copper, zinc sulfate improved clinical symptoms significantly in the third year of patient therapy [5]. Therefore, zinc therapy is used for the maintenance phase after treatment with copper chelators. There is some indication for combination therapy with copper chelators. Still, the practicality of this therapy is difficult because other copper chelators can potentially bind to zinc and decrease its effectiveness. The adverse effects are mild, including gastric irritation and the potential to induce copper deficiency.

Dietary Modifications

People with WD must follow a low-copper diet. A diet high in copper cannot cause the disease; however, if someone has a genetic disorder, it can increase the chance of clinical symptoms. Food high in copper, like lobster, liver, mushrooms, dried fruits, cocoa, and nuts, can be easily avoided in patients with WD. Other foods to avoid include beef liver, black-eyed peas, vegetable juice, and shellfish. In addition, WD patients should not take any vitamins or mineral supplements that contain copper. The practical nature of eliminating copper from the diet is challenging. It is recommended that males consume around 1.6 mg/day and females 1.3 mg/day of copper [29]. It is noted that a vegetarian diet may be a management tool for WD, and it can delay the onset of the disease and control its progression by reducing the bioavailability of copper [30]. However, the vegetarian diet should not be used as the sole therapy.

Liver Transplant

Splenectomy and orthotopic liver transplantation are the two main surgeries performed for an individual with WD. These operations are reserved for decompensated liver disease, where medication alone cannot alleviate symptoms. It is important to note that even those liver transplantation increases copper excretion, it is not beneficial for patients with the neurological form of WD. Long-term neurological damage will not be corrected by liver transplantation [5].

Emerging new therapies

Cell Therapy

Restoring ATP7B-related ATPase activity at a level sufficient for a normal copper metabolism may be possible by transplantation of an appropriate number of healthy hepatocytes. Few animal studies have indicated promising results [31,32]. Interestingly, in the WD rat model, normal hepatocyte transplantation has been linked to reduced inflammation, improved short-term survival, and protection against fulminant hepatitis in the WD rat model [31]. This shows promising results for a minimally invasive therapy that can correct both the hepatic and neurological forms of WD. Repeated cycles of cell transplantation may be required to achieve adequate clinical improvement and overcome the limitations of a single therapy session [32]. There are no ongoing reported clinical studies of cell therapies in WD patients.

ALXN1840 (Bis-choline TTM)

Another therapy that is undergoing clinical trials is the new derivative of TTM. The use of ammonium TTM and bis-choline TTM are in their second and third phase in clinical trials with the potential to be the upcoming first-line therapy of WD [23]. Bis-choline TTM has been shown to have a higher affinity for copper than the standard D-penicillamine treatment, with fewer side effects reported. Due to its extremely high binding affinity to copper, mitochondrial integrity and blood-brain barrier integrity were preserved [33].

Gene Therapy

Gene therapy is a treatment that involves introducing therapeutic genes into specific cells to replace faulty or missing genes or supplement existing genes with additional functional copies. This treatment approach has been studied in clinical trials, and the hope is that it will restore normal gene function and alleviate the symptoms of the disease. Most gene therapies involving an adeno-associated virus (AAV) are experimental, which means that clinical studies are still being conducted to determine their long-term effects and safety.

Gene therapy with recombinant AAV to deliver a functional *ATP7B* gene (cDNA) holds promise for a potential treatment for WD. The challenge associated with packing full-length *ATP7B* cDNA in the AAV gene therapy vector and low virus production yield [34] has resulted in the development of miniature *ATP7B* (mini*ATP7B*) by deleting the first four metal binding domains from *ATP7B* [35,36]. This product has been shown in vitro to have *ATP7B* activity [35,36]. The effectiveness of adeno-associated vector encoding human *ATP7B* cDNA variants in a mouse model of WD [34,36-38] in restoring copper metabolism has led to the evaluation of the two gene therapies, UX701 and VTX-801, for the treatment of WD in human studies (NCT04537377; NCT04884815).

UX701 is an investigational AAV9 gene therapy designed to deliver a modified form of the *ATP7B* transgene to produce normally functioning ATP7B protein. The US FDA has granted the product orphan drug designation. It is being evaluated in a phase 1/2/3 study to determine the safety and efficacy of a single intravenous infusion of UX701 on copper regulation in WD patients (NCT04884815). The primary outcome measured will be a change in total copper, non-ceruloplasmin-bound copper, and ceruloplasmin activity from baseline at 52 weeks. In addition, the measurement will include changes in 24-hour urinary and liver copper concentrations assessed by liver biopsy from baseline at 52 weeks. The WD patients who will be receiving UX701 will be receiving prophylactic oral corticosteroids also (NCT04884815).

VTX-801 is an investigational rAAV-based gene therapy for WD that received a US FDA fast-track designation. It is designed to deliver a miniaturized *ATP7B* transgene encoding a functional protein that has been shown to restore copper homeostasis, reverse liver pathology, and reduce copper accumulation in the brain of a WD mouse model. VTX-801 is being evaluated in a phase 1/2 clinical trial to determine a single intravenous infusion's safety, tolerability, and pharmacological activity in adult WD patients for up to five years (NCT04537377), before and following background WD therapy withdrawal. Additionally, endpoints that will be evaluated include changes in disease-related biomarkers, including free serum copper and serum ceruloplasmin activity, as well as radiocopper-related parameters.

Discussion

Though WD is recognized as a hepatocellular and neurological disease, the clinical manifestations and pathophysiology behind the specific symptoms seen with the neurological forms of WD are still gray area. When disrupting neurons in the brain and specifically the striatum, there is a great magnitude of symptoms that overlap with other neurological diseases. To correctly diagnose WD, clinicians need to recognize the most prominent forms of the disease presentation, like a wing-beating tremor, dysarthria, dysphagia, cerebellar ataxia, parkinsonism, and psychiatric manifestations.

Following the proper diagnostic outline emphasizing taking a good history and neurological examination should clue physicians in for keeping a differential of WD. The next step for proper diagnosis is labs to look for a decrease in the patient's ceruloplasmin, a disease hallmark. Keiser-Fisher rings can be seen on slit lamp examination, and MRI can show the panda sign when hyperintense signals are displayed around the midbrain, red nucleus, and substantia nigra [20]. These diagnostics criteria can help confirm the clinician's suspicion of WD.

Treating the neurological form of WD is treating not only the disease underlying metabolic pathogenesis but also each specific symptom of the neurological form of WD. For example, it is important to treat dystonia with anticholinergics while treating the biological process of WD with TTM. It is shown that chelation therapy can stop disease progression in WD. Still, physicians should carefully review each drug to determine which type of therapy best fits their patients. Overall, understanding the symptoms of the neurological form of WD will allow physicians to keep WD in their differential when a patient comes in with neurological forms of the disease. Diagnostic modalities explained will give the confirmatory diagnosis and treatment of the symptom and underlying disease process, which is important for better disease outcomes.

## Conclusions

The neurological form of WD is a well-documented disorder involving the downregulation of the *ATP7B* gene leading to an excess copper buildup within the liver, brain, and kidney. The early recognition of disease characteristics of the neurological-type WD will allow clinicians to make an early and accurate diagnosis and initiate management strategies on time. The diagnostic features of neurologic WD include Kayser-Fleischer rings, urine copper levels, serum copper levels, measured hepatic copper, "panda sign," and "bright claustrum sign." Early empiric treatment modalities with copper chelators and symptom-based management have been demonstrated to decrease symptoms and disease severity without increasing neurotoxicity and offer patients a better quality of life. Further studies and research in WD can help researchers and clinicians better understand the complexities of WD and gain an understanding of new emerging therapeutics.
